# Distribution and spatial autocorrelation of viral hepatitis B and C in Paraná, Brazil: an ecological study, 2011-2019

**DOI:** 10.1590/S2237-96222023000200015

**Published:** 2023-08-07

**Authors:** Gabriel Pavinati, Lucas Vinícius de Lima, Isadora Gabriella Silva Palmieri, Gabriela Tavares Magnabosco

**Affiliations:** 1 Universidade Estadual de Maringá, Programa de Pós-Graduação em Enfermagem, Maringá, PR, Brazil

**Keywords:** Hepatitis B, Hepatitis C, Ecological Studies, Spatial Analysis, Hepatitis B, Hepatitis C, Análisis Espacial, Estudios Ecológicos, Hepatite B, Hepatite C, Estudos Ecológicos, Análise Espacial

## Abstract

**Objective:**

to analyze the distribution and spatial autocorrelation of hepatitis B and C detection rates in the state of Paraná, Brazil.

**Methods:**

this was an ecological study of hepatitis B and C notifications held on the Notifiable Health Conditions Information System, between 2011 and 2019. Percentage change in detection rates between the first and last three-year periods was estimated. Spatial autocorrelation was analyzed using Moran’s index.

**Results:**

there were 16,699 notifications of hepatitis B, with a greater reduction in detection in the North (-30.0%) and Northwest (-25.9%) macro-regions. There were clusters of high occurrence in the Foz do Iguaçu, Francisco Beltrão and Cascavel regions between 2011 and 2019. There were 10,920 notifications of hepatitis C, with a greater reduction in detection in the Northwest macro-region (-18.9%) and an increase in the West (51.1%). The Paranaguá region recorded a high detection cluster between 2011 and 2016.

**Conclusion:**

hepatitis B and C showed heterogeneous distribution between health regions.

## RESUMO

**Objetivo:**

analisar a distribuição e a autocorrelação espacial das taxas de detecção das hepatites B e C no estado do Paraná, Brasil.

**Métodos:**

estudo ecológico das notificações de hepatites B e C no Sistema de Informação de Agravos de Notificação, 2011-2019; estimou-se a variação percentual das taxas de detecção entre o primeiro e o último triênios do período; analisou-se a autocorrelação espacial pelo índice de Moran.

**Resultados:**

houve 16.699 notificações de hepatite B, com maior diminuição da detecção nas macrorregionais de saúde Norte (-30,0%) e Noroeste (-25,9%) paranaenses; foram observados *clusters* de alta ocorrência nas regionais de saúde de Foz do Iguaçu, Francisco Beltrão e Cascavel (2011-2019); para hepatite C, houve 10.920 notificações, com maior redução da detecção na macrorregional Noroeste (-18,9%) e aumento na Oeste (+51,1%); a regional de Paranaguá registrou *cluster* de alta detecção (2011-2016).

**Conclusão:**

as hepatites B e C apresentaram distribuição heterogênea entre regionais de saúde.

## RESUMEN

**Objetivo:**

analizar la distribución y autocorrelación espacial de tasas de detección de hepatitis B y C en el estado de Paraná, Brasil.

**Métodos:**

estudio ecológico de notificaciones de hepatitis B y C en el Sistema de Información de Enfermedades de Declaración Obligatoria, entre 2011 y 2019. Se estimó la variación porcentual de las tasas de detección entre el primer y el último trienio del período. La autocorrelación espacial se analizó por el índice de Moran.

**Resultados:**

hubo 16.699 notificaciones de hepatitis B, con mayor reducción de detección en las macrorregiones Norte (-30,0%) y Noroeste (-25,9%). Hubo conglomerados de alta ocurrencia en regiones de Foz do Iguaçu, Francisco Beltrão y Cascavel entre 2011 y 2019. Para la hepatitis C, hubo 10.920 notificaciones, con una mayor reducción en la detección de la macrorregional Noroeste (-18,9%) y aumento en la Oeste (51,1%). La regional de Paranaguá registró un conglomerado de detección alto entre 2011 y 2016.

**Conclusión:**

la Hepatitis B y C mostraron distribución heterogénea entre regionales de salud.

**Table:** 

Study contributions	
Main results	There was heterogeneity in the distribution of hepatitis B and C detection rates between the Paraná health regions, with higher hepatitis B detection rates in the West health macro-region and higher hepatitis C detection rates in the East and West macro-regions.
**Implications for services**	The analysis showed the existence of priority areas for targeting strategies for hepatitis detection and prevention, service planning and organization, regarding hepatitis B and C case management in Paraná.
**Perspectives**	It is necessary to strengthen Primary Health Care performance, through the training of health professionals at this level of care, with a view to eliminating hepatitis B and C by 2030.

## INTRODUCTION

Viral hepatitis is an infectious disease that affects millions of people around the world. Hepatitis B and C, caused by viruses type B (HBV) and C (HBC), respectively, cause the majority of deaths attributed to hepatitis.^1^ Both hepatitis B and C are chronic conditions and share the same transmission routes (horizontal and vertical). HBV is mostly transmitted through unprotected sexual intercourse, while HCV is mostly transmitted through blood transfusion and needle sharing.^1^

Even today, these diseases represent an important international public health problem and have gained greater prominence on the global agenda with the establishment of goals for their elimination, proposed by the World Health Organization (WHO) and included in the Sustainable Development Goals (SDGs). Among these goals, a reduction of 90% in hepatitis B and C incidence and 65% in their mortality by 2030 is envisaged.^2^ ^,^ ^3^

In Brazil, more than 670,000 cases of viral hepatitis were reported between 1999 and 2019, with higher occurrence of hepatitis B and C. In 2019, the southern region of the country had the highest detection rates for hepatitis B (15.1/ 100,000) and hepatitis C (23.9/100,000), with the state of Paraná having the sixth highest rate for hepatitis B (15.5/100,000) and the third highest for hepatitis C (12.2/100,000), surpassing the national averages of 6.6/100,000 for hepatitis B and 10.8/100,000 for hepatitis C.^4^

Brazil’s epidemiological indicators show large regional disparities, a possible reflection of differences in achievement of follow-up measures, control and management of infectious diseases between states and municipalities.^5^ ^,^ ^6^ Differences in basic sanitation infrastructure conditions and in the organization of the health care network, as well as care service provision, contribute to the heterogeneity of health indicators.^5^ ^,^ ^6^

Early diagnosis is essential for addressing hepatitis, either to interrupt the transmission chain or to prevent progression to chronic disease.^7^ However, it is necessary to consider that response capacity and epidemiological surveillance are influenced by the geopolitical context, pointing to the importance of studies that analyze spatial distribution in order to help the understanding of the epidemiological scenario and the control of these communicable diseases.^8^

In this sense, it is necessary to recognize the locoregional singularities that support the epidemiology of these infectious diseases, in order to achieve adequate control of them. It is also worth checking the possible repercussion of actions to address the COVID-19 pandemic, carried out by health care and surveillance services in the state of Paraná, which could cloud the real epidemiological scenario of viral hepatitis.^9^

Considering that hepatitis B and C are an international priority on the 2030 Agenda, and recognizing the potential of spatial analyses for surveillance of transmissible conditions and strategic planning of actions to address them, this study aimed to analyze the distribution and spatial autocorrelation of viral hepatitis B and C detection rates in the state of Paraná, Brazil, from 2011 to 2019.

## METHODS

This was an ecological study having as its units of analysis the health regions of the state of Paraná. According to the Brazilian Institute of Geography and Statistics (Instituto Brasileiro de Geografia e Estatística - IBGE), the state of Paraná, located in the southern region of the country, has a territorial extension of 199,298.981 km², 399 municipalities and an estimated population of over 11 million inhabitants, being the most populous state in the southern region.^10^

Health service configuration in the state follows the organizational-systemic model, comprising the following administrative-sanitary territorial distribution: four health macro-regions, subdivided into 22 health regions, sharing common demographic and epidemiological characteristics,^11^ with a view to decentralization of actions and services.

The data that served as the basis for the study were derived from the Notifiable Health Conditions Information System (Sistema de Informação de Agravos de Notificação - SINAN) and the IBGE, accessed via the Brazilian National Health System Information Technology Department (DATASUS) on October 28, 2022. The study was based on cases with etiological classification as hepatitis B and C, without defining age groups, according to year of diagnosis and health region of residence, for the period from 2011 to 2019. Cases with unknown and/or blank etiological classification at the time of notification were discarded.

The viral hepatitis detection rates were calculated by dividing the number of cases of viral hepatitis, in a given period and place of residence, by the population in the same place and period, and then multiplying the result by 100,000 inhabitants. This calculation was performed for each health macro-region and region, according to pre-established three-year periods, namely: 2011-2013, 2014-2016 and 2017-2019.

The percentage change in rates between the first and last three-year periods was calculated by subtracting the last period rate from the first period rate, dividing the result by the first period rate, and then multiplying the result of this by 100. In order to analyze the spatial distribution of the detection rates, we produced maps with QGIS® software version 2.36, using the Paraná shapefile obtained from the Ministry of Health’s Open Data Portal.

We built thematic maps of hepatitis B and C for each three-year period, based on the rate intervals classified by the natural breaks technique, as proposed by Jenks. Regions with higher detection rates were represented by darker colors, while those with lower rates were represented by lighter colors, in order to keep regions with close values in the same interval and discrepant regions in different classifications.

We then analyzed detection rate spatial autocorrelation using Moran statistics, subdivided into the global Moran’s index (I) and the local Moran’s index (Ii). This analysis was performed to measure the relationship between detection rates and their spatial proximity. Initially, univariate global statistics were applied, based on the first-order queen-type neighborhood criterion.^12^

The global Moran’s index value ranges from -1.00 to +1.00: values close to 0.00 indicate spatial randomness; and values close to 1.00 indicate the presence of spatial autocorrelation, which can be direct (+) or inverse (-). In order to verify the significance of I, we applied the pseudo-significance test with 999 permutations; and when the value of I was significant (p-value < 0.05), Ii was applied (also called the Local Indicator of Spatial Association, or LISA).^12^

The Ii value was verified for each region, in order to recognize significant clusters (p-value < 0.05), categorized as follows: high-high (HH), when health regions and their neighbors had high rates; low-low (LL), when regions and their neighbors had low rates; low-high (LH), for regions with low rates and neighbors with high rates; high-low (HL), for regions with high rates and neighbors with low rates; and not significant (NS), when there was no clear spatial trend.^12^ We used GeoDa® version 1.12, and QGIS® version 2.36.

The research project was approved by the Universidade Estadual de Maringá Research Ethics Committee, in accordance with National Health Council Resolution No. 466, dated December 12, 2012, by means of Opinion No. 5.721.740, issued on October 25, 2022. It should be noted that, as this study used aggregated and non-nominal data, it was exempt from having to use an Informed Consent Form.

## RESULTS

**Figure 1:**
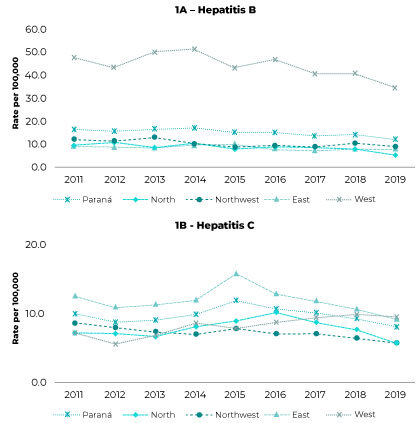
- Hepatitis B (A) and C (B) dectection rate time series, per 100,000 inhab., by Paraná health macro-regions, 2011-2019

Between 2011 and 2019, 16,699 hepatitis B cases were reported in the state of Paraná, with higher detection rates being found in the West health macro-region, throughout the entire time series. With regard to hepatitis C, 10,920 cases were reported in the same period, with higher detection rates being identified in the East health macro-region; the exception, in the case of hepatitis C, was 2019, when the detection rates for the West macro-region were higher than those found for the East macro-region (Figure 1).

A decrease in hepatitis B detection rates was found in all health macro-regions, when estimating the percentage change between the beginning and the end of the time series, in particular in the North macro-region (-30.0%), followed by the Northwest (-25.9%), West (-16.5%) and East (-15.7%) macro-regions. Despite the drop in detection rates in all macro-regions, there was an increase in the 4^th^ (+27.8%), the 18^th^ (+22.4%), the 19^th^ (+10.0%) and the 5^th^ (+0.8%) health regions (Table 1).

**Table 1:** - Hepatitis B detection rate distribution (per 100,000 inhab.), by Paraná health macro-regions and health regions, 2011-2019

Health macro-region/region	2011-2013		2014-2016		2017-2019		PC^b^
	N	Rate^a^	N	Rate^a^	n	Rate^a^	
East	1,662	10.6	1,582	9.8	1,489	9.0	-15.7
1^st^ Paranaguá	63	7.6	52	6.1	53	6.0	-20.9
2^nd^ Metropolitana	1,292	12.8	1,254	12.0	1,132	10.4	-18.4
3^rd^ Ponta Grossa	84	4.7	94	5.1	84	4.4	-5.4
4^th^ Irati	18	3.6	19	3.7	24	4.6	+27.8
5^th^ Guarapuava	168	12.4	133	9.8	171	12.5	+0.8
6^th^ União da Vitória	23	4.5	12	2.3	11	2.1	-53.7
21^st^ Telêmaco Borba	14	2.6	18	3.3	14	2.5	-4.3
West	2,869	50.3	2,905	50.1	2,479	42.0	-16.5
7^th^ Pato Branco	449	58.1	450	57.3	388	48.6	-16.3
8^th^ Francisco Beltrão	666	63.9	590	55.9	590	55.1	-13.7
9^th^ Foz do Iguaçu	504	42.2	535	44.5	422	34.9	-17.4
10^th^ Cascavel	747	47.5	922	57.3	634	38.6	-18.6
20^th^ Toledo	503	45.0	408	35.4	445	37.6	-16.4
Northwest	792	14.7	598	10.9	611	10.9	-25.9
11^th^ Campo Mourão	187	18.4	100	10.0	133	13.4	-27.1
12^th^ Umuarama	121	14.8	82	10.0	79	9.6	-35.7
13^th^ Cianorte	87	19.5	42	9.1	60	12.6	-35.5
14^th^ Paranavaí	53	6.6	66	8.1	54	6.5	-0.7
15^th^ Maringá	344	14.9	308	12.9	285	11.5	-23.2
North	662	11.5	572	9.8	478	8.1	-30.0
16^th^ Apucarana	141	13.0	67	6.0	86	7.5	-42.3
17^th^ Londrina	382	14.1	364	13.0	269	9.4	-33.3
18^th^ Cornélio Procópio	46	6.7	51	7.5	55	8.2	+22.4
19^th^ Jacarezinho	35	4.1	36	4.2	39	4.5	+10.0
22^nd^ Ivaiporã	58	13.9	54	13.4	29	7.4	-46.6

a) Rate = rate per 100,000 inhab.; b) PC = percentage change.

**Figure 2:**
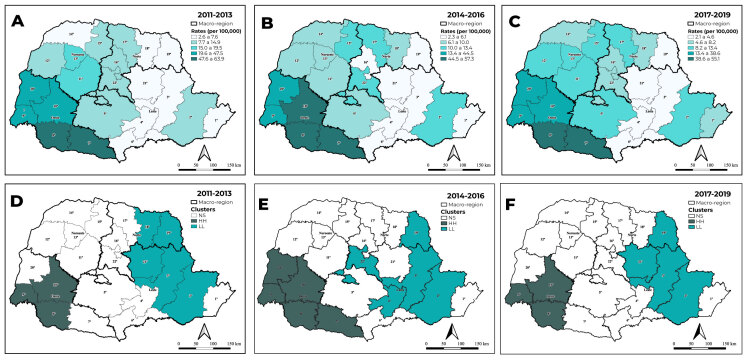
- Hepatitis B detection rate distribution (A, B and C) and spatial autocorrelation (D, E and F), per 100,000 inhab., by Paraná health regions, 2011-2019

The spatial distribution analysis showed higher hepatitis B detection rates in the West health macro-region, in all periods, in particular in the 7^th^, 8^th^ and 10^th^ health regions. The analysis of spatial autocorrelation using the univariate global Moran’s index showed significant spatial autocorrelation for the periods 2011-2013 (I = 0.639; p-value < 0.001), 2014-2016 (I = 0.645; p-value < 0.001) and 2017-2019 (I = 0.635; p-value < 0.001) (Figures 2A, 2B and 2C).

LISA was therefore used to produce maps with significant clusters (p-value <0.05). A high-high spatial autocorrelation was identified for the 8^th^, 9^th^ and 10^th^ health regions, over the three three-year periods, expanding to all health regions of the West macro-region in the second three-year period. In turn, autocorrelation of a type categorized as low-low was found in the health regions of the North and East macro-regions, in the three three-year periods (Figures 2D, 2E and 2F).

With regard to hepatitis C, the percentage change in the detection rate between the first and last three-year periods showed a reduction in the East (-6.6%) and Northwest (-18.9%) health macro-regions, and an increase in detection in the West (+51.1%) and North (+4.5%) health macro-regions. The biggest drops were found in the 15^th^ (-23.9%), 22^nd^ (-19.8%) and 14^th^ (-18.0%) health regions, while the largest increases were identified in the 10^th^ (+80.0%), 18^th^ (+47.0%) and 5^th^ health regions (+45.8%) (Table 2).

**Table 2:** - Hepatitis C detection rate distribution (per 100,000 inhab.), by Paraná health macro-regions and health regions, 2011-2019

Health macro-region/region	2011-2013		2014-2016		2017-2019		PC^b^
	N	Rate^a^	N	Rate^a^	n	Rate^a^	
East	2,084	13.3	2,298	14.3	2,068	12.5	-6.6
1^st^ Paranaguá	107	12.9	147	17.2	160	18.1	+40.5
2^nd^ Metropolitana	1,654	16.4	1,834	17.5	1,561	14.4	-12.1
3^rd^ Ponta Grossa	195	10.9	190	10.3	182	9.6	-11.7
4^th^ Irati	17	3.4	16	3.1	21	4.0	+18.4
5^th^ Guarapuava	53	3.9	52	3.8	78	5.7	+45.8
6^th^ União da Vitória	18	3.5	14	2.7	20	3.8	+7.5
21^st^ Telêmaco Borba	40	7.4	45	8.2	46	8.2	+10.0
West	399	7.0	511	8.8	624	10.6	+51.1
7^th^ Pato Branco	45	5.8	65	8.3	59	7.4	+27.0
8^th^ Francisco Beltrão	39	3.7	35	3.3	50	4.7	+24.9
9^th^ Foz do Iguaçu	116	9.7	142	11.8	167	13.8	+42.1
10^th^ Cascavel	138	8.8	186	11.6	259	15.8	+80.0
20^th^ Toledo	61	5.5	83	7.2	89	7.5	+37.8
Northwest	514	9.5	488	8.9	434	7.7	-18.9
11^th^ Campo Mourão	66	6.5	53	5.3	57	5.8	-11.5
12^th^ Umuarama	42	5.2	39	4.7	41	5.0	-3.8
13^th^ Cianorte	17	3.8	10	2.2	15	3.1	-17.5
14^th^ Paranavaí	88	10.9	101	12.4	74	9.0	-18.0
15^th^ Maringá	301	13.1	285	11.9	247	9.9	-23.9
North	447	7.8	571	9.8	482	8.1	+4.5
16^th^ Apucarana	80	7.4	85	7.6	73	6.4	-13.6
17^th^ Londrina	256	9.4	361	12.9	272	9.5	+0.6
18^th^ Cornélio Procópio	39	5.7	45	6.6	56	8.4	+47.0
19^th^ Jacarezinho	52	6.1	66	7.7	66	7.6	+25.3
22^nd^ Ivaiporã	20	4.8	14	3.5	15	3.8	-19.8

a) Rate = rate per 100,000 inhab.; b) PC = percentage change.

The spatial distribution showed that the highest hepatitis C detection rates were concentrated in the 1^st^, 2^nd^, 3^rd^, 14^th^ and 15^th^ health regions in the first three-year period, as well as the 9^th^, 10^th^ and 17^th^ health regions in the second three-year period. In the final three-year period, higher hepatitis C detection rates were observed in the East macro-region (1^st^ and 2^nd^ health regions) and the West macro-region (9^th^ and 10^th^ health regions), with a fall in rates in the 2^nd^, 3^rd^, 14^th^, 15^th^ and 17^th^ health regions.

**Figure 3:**
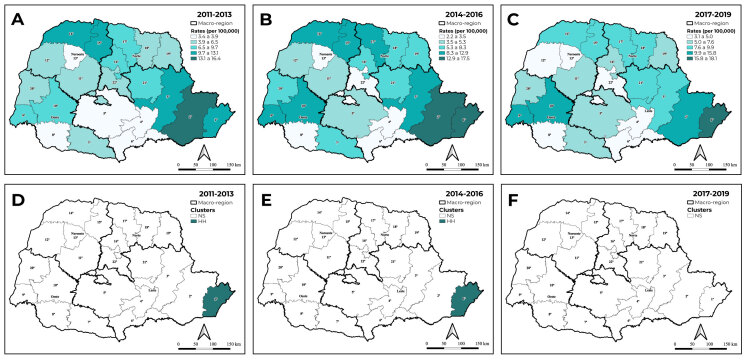
- Hepatitis C detection rate distribution (A, B and C) and spatial autocorrelation (D, E and F), per 100,000 inhab., by Paraná health regions, 2011-2019

The univariate global Moran’s index showed significant spatial autocorrelation in the periods 2011-2013 (I = 0.222; p-value = 0.037), 2014-2016 (I = 0.220; p-value = 0.028) and 2017-2019 (I = 0.208; p-value = 0.032) (Figure 3A, 3B and 3C). A high-high autocorrelation was identified in the first two three-year periods only for the 1^st^ health region, located in the East health macro-region. Detection rates for the final three-year period did not reveal significant spatial autocorrelation in any of Paraná’s health regions (Figures 3D, 3E and 3F).

## DISCUSSION

Analysis of the percentage change in hepatitis B detection rates showed a reduction in all health macro-regions in the state of Paraná, with the exception of some health regions in the North and East macro-regions. As for detection rate spatial distribution, the analysis showed higher hepatitis B detection rates in the West macro-region, where a high-high spatial autocorrelation was found, and a low-low autocorrelation in some health regions of the North and East macro-regions.

With regard to hepatitis C, higher detection rates were found in the East health macro-region, which presented a high-high spatial autocorrelation in the 1^st^ health region, Paranaguá. Analysis of percentage change in rates identified an increase in detection in the West and North macro-regions, and a reduction in detection in the East and Northwest macro-regions. It should be noted that all the health regions in the West macro-region showed an increase, while all those in the Northwest macro-region showed a decrease.

The decrease observed in the number of hepatitis B cases, in the vast majority of health regions in Paraná, may be related to actions to address this disease proposed by the Brazilian Ministry of Health, such as increasing vaccination coverage, reducing vertical transmission and distributing supplies and medicines for prevention, testing and treatment,^4^ these being actions that contribute to prevention, early diagnosis and timely treatment and, consequently, interruption of the transmission chain.

Despite the generalized drop in hepatitis B, it should be noted that some health regions showed a significant increase in the number of cases, possibly related to the increase in virus circulation or the expansion of availability and/or access to diagnosis, due to the use of tests for investigation in various population segments with effect from 2015.^13^ This is a finding that points to the need to carry out studies aimed at understanding local realities and explaining these results.

The concentration of hepatitis B detection rates in the West health macro-region of the state corroborates the results of a study that showed the existence of high-high type clusters in the municipalities of this macro-region, denoting that the condition still represents an important public health problem in border regions, despite the availability of vaccines, diagnostic tests and treatment.^14^

It should be mentioned that the concentration of cases in a region close to the so-called Three Frontiers (Típlice Fronteira) - between the state of Paraná, Paraguay and Argentina - is in line with a study carried out in the Northern region of Brazil, where higher occurrence of viral hepatitis was found in areas that border with other South American countries, especially those with a high infection burden.^15^

It is known that border regions sustain greater socioeconomic disparities and, generally being marginalized, suffer from the impact of this condition on the health of the population of the respective countries.^16^ It is necessary to formulate and implement internationally articulated policies, for their adequate local development in border regions, overcoming legal asymmetries.^17^

Furthermore, there is a lack of initiatives that consider the cross-border process as a phenomenon that weakens access to health services, making it unstable.^17^ Indeed, it is perceived as a great challenge for epidemiological surveillance, health manager and health service performance, and the need for an intersectoral, holistic and collaborative approach between the countries that border the state of Paraná.

Moreover, there was a higher concentration of hepatitis C cases in health regions of the East and West macro-regions, pointing to possible regional disparities and the need to define coping strategies focused on these regions. Hepatitis C continues to be a health challenge, demanding improvements in access to diagnosis and treatment, with a view to eliminating the condition.^18^

According to the Ipardes Municipal Performance Index (Índice Ipardes de Desempenho Municipal - IPDM), the objective of which is to estimate the socioeconomic conditions of Paraná’s municipalities, the East Paraná health macro-region concentrates most of the cities with the lowest performance according to the index, in terms of income, education and health aspects, among others.^19^ This issue may be linked to the persistent concentration of the highest number of hepatitis C cases in that macro-region.

The findings of the present study highlight the need for efforts to combat viral hepatitis, especially in regions with greater virus circulation/detection, through the implementation of immunization strategies, early diagnosis and effective treatment, with a view to overcoming the current scenario.^14^ In order for the elimination of the problem to be sucessful, the need for strategies at the global and local level must be considered, in accordance with the epidemiology of the disease.^20^

Beyond the aforementioned strategies for addressing the disease, the need for health education for the population stands out, especially in the highlighted priority regions, guided by the singularities of each region, with the objective of developing assertive and effective actions towards the elimination of the condition, at both the regional level and, in the end, throughout Paraná as a whole.^21^ ^,^ ^22^

It should be noted here that a previous study identified geographic inequalities in relation to the distribution of infectious diseases in the state’s health regions, which may explain the local disparities in the population’s health conditions.^23^ Hence the importance of the findings related to hepatitis B and C and the identification of priority regions with a high disease burden, for the implementation of local actions targeting these diseases.

 In addition, there is a need for studies that delve deeper into the management processes within the regional health departments, which are sometimes subject to political guidelines and priority criteria that are unrelated to those of the state as a whole. Due to the Chronic Conditions Care Model (Modelo de Atenção às Condições Crônicas - MACC), Paraná has increasing planning of agreements and goals, which starts from the municipalities towards the state as a whole, which can influence the effectiveness and prioritization of actions developed at the local level.^24^ ^,^ ^25^

With regard to health care for viral hepatitis, some advances that have taken place in recent years are worth highlighting, such as the decentralization of treatment of liver infections caused by viruses to Primary Health Care, in addition to other actions already developed.^26^

Still with regard to the potential of Primary Health Care, it is fair to publicly recognize the role of nursing professionals in addressing viral hepatitis.^27^ Given the role of nurses and other professionals in care, teaching and research, there is a possibility of greater qualification of strategies aimed at the control and management of these conditions based on health care with an emphasis on comprehensive services and articulation between their different stakeholders, based on surveillance of the condition, in order to expand access to care beyond specialized services.^15^

We suggest that health service managers and professionals in Paraná structure the organization of services and work processes, taking into account the strengthening of Primary Health Care and the role of nursing professionals with regard to actions to combat hepatitis B and C. The development of lines of care and the design of care flowcharts can contribute to the interaction of services and the provision of comprehensive care, this being a principle of the Brazilian National Health System. 

One of the limitations of this research, in addition to those related to the use of secondary data that are subject to incomplete and/or erroneous filling in, is the fact that the variation in detection rates in the period analyzed can be linked to individual factors, such as greater perception of risk and demand for services, and/or programmatic factors related to the availability of and access to diagnostic tests, which, possibly, would also explain the differences seen in the distribution of viral hepatitis cases in the health regions.

In short, the study showed that hepatitis B and C did not have homogeneous distribution in Paraná’s health regions. There was a predominance of hepatitis B cases in the West health macro-region, with a drop in detection in most of the state’s health regions and the presence of high-risk clusters, especially in the Foz do Iguaçu, Francisco Beltrão and Cascavel regions, in the three three-year periods analyzed.

Regarding the hepatitis C detection rates, the cases were concentrated in the East and West health macro-regions of the state, with both an increase and a decrease in the percentage change between regions. A weak spatial autocorrelation was also seen by using Moran’s index. A cluster of high occurrence was identified in the Paranaguá health region, in the first two three-year periods analyzed.

There is therefore a need for policies and coping strategies that consider local singularities and guarantee the assertive targeting of actions and services aimed at preventing and caring for viral hepatitis in the state of Paraná. The importance and potential of epidemiological surveillance is highlighted, as a guiding instrument for hepatitis B and C management and care, supporting the planning of actions and the organization of services, especially for the areas with the highest occurrence of these diseases.
